# From Pilot to Practice: A Sociotechnical Perspective for Sustainable Adoption of Patient Engagement Technologies

**DOI:** 10.1055/a-2843-8762

**Published:** 2026-04-21

**Authors:** Prashila Dullabh, Courtney Zott, Nicole Gauthreaux, Abigail Aronoff, Dean F. Sittig, Aziz Boxwala

**Affiliations:** 1 Health Sciences Department, NORC at the University of Chicago, Washington, District of Columbia, United States; 2 Informatics Review LLC, Lake Oswego, Oregon, United States; 3 Elimu Informatics, El Cerrito, California, United States

**Keywords:** clinical decision support systems, patient engagement, sociotechnical aspects of information technology, implementation and deployment, standards adoption

## Abstract

**Background:**

Despite widespread investment in patient engagement technologies—such as mobile apps, chatbots, and remote monitoring tools—few have achieved sustained adoption or integration into clinical workflows. The persistent gap between pilot success and real-world scalability reflects not only technical barriers but also sociotechnical challenges involving people, processes, and policy.

**Objectives:**

This study aimed to identify cross-cutting barriers and enablers of implementation across multiple real-world pilots of patient engagement technologies and extend the Sociotechnical Model (STM) of Health Information Technology (IT) to explicitly incorporate patient perspectives and lived experiences as determinants of adoption and sustainability.

**Methods:**

Drawing on our team's formal evaluations of implementing patient engagement technologies across four U.S. health systems—including applications for coronavirus disease 2019, hypertension, medication adherence, and chatbot-supported communication—we synthesized lessons learned across eight STM domains: hardware/software, clinical content, human–computer interface, people, workflow and communication, organizational policies, external pressures, and system monitoring.

**Results:**

Eight cross-cutting lessons emerged: (1) effective leadership and collaboration across clinicians, IT and informatics teams, patients, and electronic health record and app developers are essential; (2) uneven standards adoption and support continues to limit interoperability; (3) success depends on skilled technical resources with expertise in interoperability standards; (4) engage patients in codesign processes early and throughout; (5) sustained patient engagement requires structured onboarding and feedback loops; (6) account for diverse patient needs and preferences during the design; (7) clinician workflows must be redesigned to integrate and act on patient-contributed data without increasing burden; and (8) demonstrated return on investment is needed to justify long-term costs of maintenance.

**Conclusion:**

Sustaining patient engagement technologies requires expanding the sociotechnical lens to include patients' lifeflows alongside organizational and technical factors. Future implementation, research, and policy efforts should focus on collaborative leadership models, patient-centered engagement processes, enhanced interoperability, clear data monitoring workflows, and financial sustainability.

## Introduction

Despite the proliferation of patient engagement technologies—such as mobile applications, chatbots, and remote monitoring tools—relatively few have achieved sustained adoption or have scaled beyond pilot implementations. 1 2 Health systems and researchers continue to invest substantial resources in developing and testing these technologies, yet the evidence base reveals a persistent gap between proof-of-concept pilots and integration into routine clinical workflows with documented improvements in patient health and provider efficiency, or reduction in costs. 3

This challenge is salient given the current federal interest in building a robust digital health ecosystem that empowers patients and clinicians. 4 While there is strong policy momentum toward digital transformation, realizing its full potential requires understanding the sociotechnical factors that shape adoption and scalability. 5 The Sociotechnical Model (STM) of Health Information Technology (IT) developed by Sittig and Singh 6 underscores that technical feasibility alone is insufficient; people, process, and policy dynamics are also critical determinants of adoption and effectiveness.

In this perspective article, we draw on our collective experience implementing and evaluating multiple real-world pilots of patient engagement technologies across diverse health systems to identify recurring implementation barriers and enablers, highlight promising strategies for future sustainability, and identify knowledge gaps that warrant further investigation. Our team—which includes clinical informaticians and implementation scientists—has worked closely with electronic health record (EHR) and app developers, clinicians, and health system leaders on initiatives directed at coronavirus disease 2019 (COVID-19), hypertension, hypertensive disorders of pregnancy (HDP), medication adherence, and chatbot-supported communication.

**Fig. 1:**
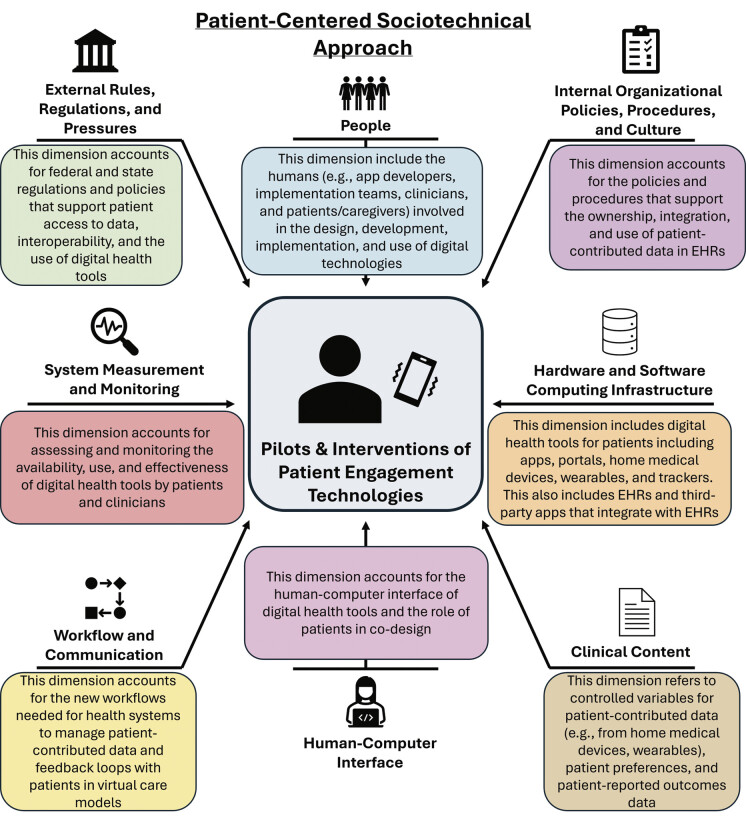
Diagram of a patient-centered sociotechnical approach for patient engagement technologies.

Our unique contribution is applying a patient-centered lens to the STM, explicitly elevating patient perspectives and experiences as key to identifying barriers, enablers, and strategies for sustainable implementation ( Fig. 1 ). While the STM traditionally focuses on technology use within health care delivery systems, we extend this framework to account for how patients engage with technologies in their everyday lives, outside of clinical settings. A patient-centered sociotechnical approach also accounts for the settings in which digital technologies get implemented and the people (e.g., clinicians, informaticians, operational leaders) and processes that are needed to ensure the technology works in service of patients' needs. By integrating patients into the sociotechnical lens, our goal is to advance both the science and implementation of patient engagement technologies in ways that ensure future efforts are responsive to patients' needs, preferences, values, and lifeflows—not solely an organization's operational or nation's policy priorities.

## Methods

The findings presented in this manuscript were informed by mixed methods evaluations conducted across multiple pilot projects. Each pilot underwent a structured evaluation with clearly defined assessment objectives, incorporating rigorous qualitative data collection from key stakeholders, including patients, clinicians, and implementation teams, as well as quantitative analyses of application usage and/or EHR data. Each evaluation resulted in a comprehensive assessment report and, in some cases, a peer-reviewed manuscript summarizing key findings and themes.

For the present analysis, we conducted a qualitative thematic synthesis of all pilot evaluation reports. Using thematic analysis, 7 we systematically reviewed the reports to distill shared lessons across pilots. Identified lessons learned were then categorized within the eight STM domains—hardware and software, clinical content, human–computer interface, people, workflow and communication, organizational policies, external pressures, and system monitoring. This synthesis was iteratively refined through team deliberations and informed by guidance from P.D. and D.F.S., ensuring analytic rigor, consistency, and alignment across evaluations.

## Pilot Experience

We have conducted a series of pilots across multiple health systems over the past 5 years to evaluate the design, implementation, and feasibility of patient engagement applications leveraging Substitutable Medical Applications and Reusable Technologies (SMART) on Fast Healthcare Interoperability Resources (FHIR) standards and other digital technologies ( Table 1 ).

**Table 1:** Project team pilot experience and characteristics

Site	Patient population/condition	Patient-facing component of technology	Patient-contributed data	Clinician-facing component of technology	Methods and data sources
Yale New Haven Health System 20	COVID-19 patients isolating at home	COVID-19 remote monitoring system	Vitals and symptom data	EHR-proprietary dashboard	• Weekly meeting notes on design and implementation ( *technical team* ) • KIIs on implementation/usability ( *6 care coordinators* )
Yale New Haven Health System 11	Postpartum patients with HDP	SMART on FHIR HDP app	BP readings and symptom data	EHR-proprietary dashboard	• Weekly meeting notes on design and implementation ( *technical team* ) • KIIs on implementation ( *2 technical team members, 4 clinicians* ) • KIIs on usability ( *2 patients* ) • EHR and app usage data
Emory Healthcare 7	Postpartum patients with HDP	SMART on FHIR HDP app	Symptom data	EHR-proprietary dashboard	• Weekly meeting notes on design and implementation ( *technical team* ) • KIIs on technical/ operational feasibility ( *5 technical team members, 3 implementation leads, 1 clinical lead* ) • EHR and app usage data
Baystate Health 10 35	Patients with uncontrolled hypertension	SMART on FHIR BP and medication adherence app	BP readings and medication adherence	EHR-integrated SMART on FHIR app	• Weekly meeting notes on design and implementation ( *technical team* ) • KIIs on implementation ( *6 technical team members, 5 clinicians* ) • KIIs on usability ( *6 patients* ) • Think-aloud sessions on usability ( *4 patients* ) • EHR and app usage data • System usability questionnaire ( *8 patients* )
Vanderbilt University Medical Center 37 38	General patient population (supporting patient–clinician communication)	LLM chatbot	Health concerns and symptoms	LLM-generated summaries for clinicians	• Think-aloud usability sessions ( *5 patients* ) • System usability questionnaire (5 *patients* ) • Timeliness data from usability session (5 *patients* ) • Questionnaires of chatbot-patient interactions ( *6 clinicians* )

Abbreviations: BP, blood pressure; COVID-19, coronavirus disease 2019; EHR, electronic health record; FHIR, Fast Healthcare Interoperability Resources; HDP, hypertensive disorders of pregnancy; KIIS, key informant interviews; LLM, large language model; SMART, Substitutable Medical Applications and Reusable Technologies.

## Cross-Cutting Lessons Learned

Across our pilots, we identified eight overarching lessons learned grouped into eight sociotechnical domains that affect the long-term success of patient engagement technologies ( Table 2 ).

**Table 2:** Sociotechnical factors addressed in lessons learned from pilots

Lesson learned	Applicable sociotechnical domain(s)
Leadership and collaboration across stakeholders	• People • Internal Organizational Policies, Procedures, and Culture
Need for expanded and standardized syntactic FHIR support	• Hardware and Software Computing Infrastructure • Clinical Content
Need for skilled technical resources with expertise in new interoperability standards like FHIR	• People
Engage patients in codesign early and throughout the pilot	• Human–Computer Interface • Workflow and Communication
Sustaining engagement through feedback loops	• Workflow and Communication
Accounting for diverse patient needs and preferences	• Human–Computer Interface • Workflow and Communication
New clinician workflows for monitoring patient-contributed data	• Human–Computer Interface • Workflow and Communication • External Rules, Regulations, and Pressures
Demonstrating effects on cost, quality, and effort	• System Measurement and Monitoring

Abbreviation: FHIR, Fast Healthcare Interoperability Resources.

### Leadership and Collaboration Across Stakeholders

Implementing patient engagement technologies involves multiple stakeholders external to the health care organization, including EHR and third-party app developers, clinicians, and patients/caregivers. As a result, successful implementation depends on organizational leadership capable of coordinating beyond the health system to engage patients and external partners. This coordination is especially critical for interventions that introduce significant technical, security, or workflow complexities, such as writing data to the EHR from apps.

In all our pilots, these leaders often included an executive-level informatics leader and one or more clinical leads or champions. 8 Executive leaders played a critical role by convening all stakeholders, establishing shared project goals, and mediating key decisions to maintain alignment across technical, clinical, and patient-centered priorities. They were instrumental in optimizing internal organizational conditions for implementation, including allocating staff time and facilitating approvals such as Institutional Review Boards and Business Associate Agreements with third-party developers. On the clinical side, clinical leads and champions collaborated with the internal technical team, EHR, and third-party app developers to shape decisions regarding data access and use, anticipate workflow modifications, and resolve integration challenges, positioning them as codevelopers rather than simply advocates.

To engage patients meaningfully, organizational leadership prioritized patient involvement and actively coordinated with app developers to facilitate patient-centered codesign activities. This emphasis from leadership ensured tools were not only technically and operationally feasible but responsive to patient values, needs, and preferences.

### Need for Expanded and Standardized Syntactic and Semantic Fast Healthcare Interoperability Resources Support

Patient engagement technologies often require integration of patient-contributed data, such as data from medical devices, wearable devices, patient-reported outcomes (PROs), and patient preferences into EHR systems in ways that patients can trust will be visible to and acted upon by their care team. While all pilots demonstrated that EHR integration is technically feasible, each implementation revealed persistent challenges in writing data to the EHR.

First, although syntactic and semantic FHIR standards and implementation guides exist, their adoption remains inadequate for many types of patient-reported data, particularly non-standardized data, like patient-reported symptoms. As a result, our pilots often had to rely on proprietary workarounds or custom builds, which undermined interoperability and scalability. Second, even when these FHIR resources existed, EHR vendor adoption of FHIR was uneven. EHR products vary in their implementation of FHIR, meaning that not all relevant data types were supported at all sites. This uneven adoption created variability in how patient data could be written back to the EHR across sites. In some cases, write-back functionality proved fragile, resulting in clinicians not being able to access or use patient-contributed data within their workflows. Other site-specific technical limitations related to write-back, such as organizational firewall constraints, slowed integration, and required significant local IT expertise.

One promising approach observed in our pilots was workflow-sensitive integration, in which third-party apps were integrated with EHRs and embedded directly into clinical workflows without requiring data write-back. In some use cases, this strategy offered clinicians the same or more sophisticated ability to view and manage data while avoiding the technical complexity and resource burden of writing back data generated by patients from medical devices or PROs into the EHR. For example, in the pilot at Baystate Health, a third-party dashboard was built to summarize patient-reported data (e.g., blood pressure [BP] readings) along with relevant EHR data (e.g., current medications and test results, recent visits) and was made accessible directly from the EHR through the patient's chart.

### Need for Skilled Technical Resources with Expertise in New Interoperability Standards Like Fast Healthcare Interoperability Resources

Across several pilot implementations that involved integrating patient-reported data into clinical workflows (including at Yale New Haven Health System, Baystate Health, and Emory Healthcare), both the health system integration team and the third-party app developer brought valuable prior experience with FHIR-enabled app integrations, which helped accelerate development and deployment. The practical knowledge of FHIR application programming interfaces (APIs), flowsheet configurations, and EHR-specific requirements enabled them to anticipate challenges and implement solutions more efficiently. For health care system partners, this expertise was instrumental in coordinating efforts across departments; managing technical dependencies among the app, integration, and EHR teams; and ensuring alignment with technical workflows.

### Engage Patients in Codesign Early and Throughout the Pilot

Codesign and usability testing with patients are fundamental for developing patient engagement technologies, ensuring that apps meet user needs and are intuitive to navigate. 9 Our pilots used several approaches to codesign with patients, including key informant interviews to gather user requirements, think-aloud sessions with patients to support rapid prototyping, and patient surveys. These approaches ensured that design decisions were informed by patient needs and perspectives rather than assumptions.

We found that our pilots (Baystate Health and Vanderbilt University Medical Center) that invested in more comprehensive codesign prior to implementation, particularly with patients at the pilot sites who would be end users of the technology, improved the patient-centeredness of the design and enhanced engagement. For example, in the Baystate Health pilot, codesign activities led to revisions in the wording and tone of text messages, resulting in more positive patient feedback on message quality and ease of use during implementation. 10 Similarly, patients expressed preferences regarding timing of messages, which we incorporated into prototype designs by allowing patients to change the time they receive messages. 11 These insights underscored that technology adoption depends on how well tools integrate into daily routines and enhance communication beyond existing methods.

### Sustaining Engagement through Feedback Loops

While codesign improved initial usability, our experience revealed that it alone does not guarantee sustained engagement in real-world settings. Patients consistently expressed the need for support in their use of the technology, including onboarding, continuous monitoring, assistance if and when they encountered technical issues, and timely feedback from clinicians. 12 Feedback loops emerged as a large motivator for patient engagement, especially for digital tools supporting chronic conditions that require long-term participation. Patients reported that contributing data outside the clinical setting felt meaningful only when it resulted in actionable responses from their care team. In practice, this would require mechanisms for clinicians to acknowledge patient contributions, provide updates, and close the loop on shared information. For these pilots, clinicians used clinical judgement to determine appropriate patient follow-up, conducting outreach manually through phone calls or portal messages. Future implementations would benefit from workflows that facilitate timely and consistent patient communication.

### Accounting for Diverse Patient Needs and Preferences

Across our pilots, we partnered with multiple health care systems serving different patient populations, including individuals with COVID-19, postpartum mothers with HDP (in both Medicaid and non-Medicaid clinics), older adults with chronic hypertension, rural and urban areas, and primary care patients with a range of medical conditions. These experiences underscored the importance of accounting for patients' unique personal situations, clinical complexity, and preferences for using technology rather than assuming uniform fit across a population.

A consistent theme across pilots was variation in patients' comfort with and preference for technology-mediated communication. While many patients valued receiving text messages or interacting with a patient portal chatbot, a few expressed concerns that they might replace communication with their clinicians or strongly preferred direct communication with their clinician due to strong existing relationships. While we clarified that these tools were supplemental to their current care, a small number of patients may always prefer direct human interaction, regardless of the sophistication or effectiveness of the technology.

We also found that patients with more complex or atypical clinical needs within the populations of interest may not always be well served by standardized workflows and communication protocols. For example, some patients with chronic hypertension had more complex comorbidities or histories that required different management. Others were enrolled in concurrent care management programs with overlapping requirements and additional demands on their time. Patients with complex conditions expected the digital health technology to be more personalized to their medical history and medication use. Across pilots, we sought to balance personalization and precision with general relevance and scalability, recognizing that increasing specificity for individual patients can introduce operational and technical complexity that challenges broader implementation. Emerging approaches using artificial intelligence may help address this tension by allowing for more adaptive communication protocols and decision support without requiring unique data and workflows for each patient.

### New Clinician Workflows for Monitoring Patient-Contributed Data

Patient engagement technologies often involve the collection of patient-contributed data that must be actively monitored and acted upon in near real-time, requiring implementations to consider both clinical workflow alignment and clinician workload. Across our pilots, different approaches were tested—including in-basket alerts, EHR-native reports with flowsheet annotations, and third-party integrated dashboards with color-coded data. Clinicians consistently reported that well-designed dashboards enhanced their ability to monitor patients between visits. 13 For example, a dashboard in the Baystate Health pilot centralized key patient data—including medications, BP readings, and medical history—and allowed patients to annotate their readings, giving clinicians context for elevated values. Clinicians also valued seeing longitudinal trends in patient data, which informed more tailored management decisions.

At the same time, pilots revealed several workflow challenges. Monitoring responsibilities were often fragmented, with different clinicians and nursing staff using variable approaches to review patient data. In some cases, the absence of automated alerts required manual review, increasing workload, and the risk of missed information. Clinicians emphasized that technologies would be more valuable if they provided services that meaningfully reduced their workload, such as supporting monitoring of patient responses, flagging abnormal data, or facilitating patient follow-up—tasks that otherwise consume significant clinician time.

Overall, what emerged as most critical was ensuring that these tools presented relevant patient data in ways that fit seamlessly into existing clinician workflows.

### Demonstrating Effects on Cost, Quality, and Effort

Despite demonstrating early signs of promising clinical utility, our pilots revealed that financial sustainability remains a major barrier to scaling patient engagement technologies. 14 In our pilots, implementation costs extended beyond the initial price of developing the app to include expenses associated with EHR integration, license fees for FHIR APIs from developers, and ongoing vendor fees (particularly for text messaging-based patient engagement technologies that generated high message volumes). Clinicians and implementation leaders also emphasized the long-term costs of maintaining and updating these technologies over time even after considering reduction in costs when scaled over a larger number of app adoptions. Together, these factors created resource demands that were difficult to justify without clear reimbursement mechanisms or demonstrable return on investment.

## Discussion

While prior applications of the STM have focused primarily on technology use within clinical environments, our findings extend it to patient-centered tools used in daily life, recognizing that although many implementation challenges are longstanding, the evolution from tools that simply present information to tools that actively support patient decision-making introduces additional layers of complexity. Taken together, our pilots suggest that sustaining patient engagement technologies requires attention to:

### Collaborative Leadership Models

Leadership should be conceptualized not only in terms of organizational authority, but also in terms of capacity to coordinate across clinicians, app and EHR developers, integration teams, and patients. Future research should explore strategies to more systematically incorporate patient perspectives—including through patient and family advisory groups—to strengthen codesign, usability, and adoption. 15 Additionally, studies should examine how health systems can implement and sustain collaborative models that integrate multistakeholder input, including appropriate incentive structures, governance mechanisms, and partnership strategies. 16

### Enhanced Electronic Health Record Interoperability

Coordinated efforts by EHR developers, app developers, and health systems are needed to expand standardized FHIR support while also developing clear policies and practices for determining when data should be written back to the EHR versus when workflow-sensitive display integration is sufficient. 16 Enhancing interoperability will require institutional policies that define how third-party apps can connect with the EHR, including approval processes, security requirements, and data access rules. 17 It will also require federal guidance clarifying compliance expectations under the Health Insurance Portability and Accountability Act, 21st Century Cures Act, and related regulations for securing and sharing patient data stored outside the EHR, as well as clinician responsibilities for acting on patient-contributed data submitted through external apps. 18 19

#### Greater transparency and standardization in how EHR developers expose the technical requirements for FHIR app integrations

While FHIR standards and EHR developer-specific documentation offer a starting point for app developers, many site-specific configuration details only become apparent during implementation. 20 21 Continued progress to make integrations more predictable will require coordinated efforts across EHR and app developers and health systems. EHR developers can publish more detailed and specific documentation to reduce variability in implementation. Additionally, they can proactively identify common integration challenges and develop comprehensive checklists to help site teams navigate problem areas more effectively.

### New Processes for Sustaining Patient Engagement

To sustain engagement at scale, implementers and health systems must move beyond one-time implementation efforts toward continuous partnership with patients across the entire digital health technology lifecycle (design, development, implementation, use, and evaluation). 22 There has been limited effort to understand how digital tools fit into patients' lifeflows, and few reliable and valid means to measure meaningful engagement across the lifecycle. 23 Health systems should integrate supportive onboarding, training, and technical support processes as a standard part of clinical care to ensure that patients can easily access and use technologies over time. 24 25 Additionally, more studies are needed to understand how engagement varies across patient characteristics, including age, chronic disease status, and levels of digital literacy, to better tailor implementation strategies. Prior research indicates that patients are more likely to engage when digital tools clearly improve symptom control, monitoring, or communication with clinicians and fit seamlessly into daily life. 26 This is especially relevant for individuals with chronic conditions, who are often older adults and may have lower levels of digital literacy and digital health literacy that influence their ability to remain engaged. 27 As hybrid and virtual care models become more common, it is important to consider a patient's digital health literacy level and adapt appointment modalities and patient engagement approaches to match digital readiness. 28 29 For instance, families and caregivers may play a larger role in care processes for patients with lower health or digital literacy. 29 Ongoing coimplementation through mechanisms like patient and family advisory groups and regular review of patient engagement metrics will be key to identifying issues with technologies, understanding which patient groups are most affected, and guiding adaptations to improve inclusivity and long-term use. 30

### Workflows for Data Monitoring

Health systems need new policies and procedures that clearly define responsibilities for monitoring patient-contributed data and ensuring that data can be acted upon in a timely manner. 12 These workflows are essential not only to ensure clinical utility but also to drive patient trust that their contributions will be seen and acted upon in a timely manner. Policies should balance clinician capacity and data competence with patient expectations, ensuring that patient-contributed data enhances care rather than creating bottlenecks or gaps in communication. 12 Similar to our Baystate Health pilot, one study engaged nurses as a first-line review of patient-reported symptom data and found that one-third of medication-related issues were resolved independently by nurses. 31 This suggests that with the proper support, nurses can play a valuable role in managing patient safety concerns and determining when physician involvement is necessary. In addition, the presence of outsourced or in-house remote monitoring services raises additional workflow considerations on how patient-contributed data will be monitored, managed, and acted upon by clinical teams. 12

### Demonstrated Financial Sustainability

Demonstrating return on investment (ROI) will require further research on both clinical outcomes (e.g., reduced complications, fewer readmissions) and operational efficiencies (e.g., avoided phone calls or visits). 32 33 Reimbursement models may also be necessary to create incentives for long-term use, such as the Centers for Medicare and Medicaid remote therapeutic monitoring, which has been used for musculoskeletal and respiratory conditions. 34 Without a compelling business case that addresses the costs of implementing these apps, even technically successful patient engagement technologies may fail to be adopted into routine clinical practice. 35 36

## Conclusion

Across multiple real-world pilots, our experience reinforces that the success of patient engagement technologies depends as much on technical feasibility as it does on the sociotechnical environment in which they are deployed. The eight overarching lessons we describe represent recurring lessons that, if addressed proactively, can substantially increase the likelihood of adoption and scalability.

For implementers, these lessons underscore the importance of aligning technologies with both clinical workflows and patient lifeflows, budgeting for EHR integration and ongoing maintenance, and identifying the right organizational leaders who can drive stakeholder collaboration. For researchers, they highlight gaps in evidence, such as strategies for sustaining patient engagement over time, demonstrating long-term financial sustainability, and understanding optimal leadership and governance structures. For developers and policymakers, they point to the need for more robust standards, predictable reimbursement pathways, and business models that support long-term sustainability. Ultimately, bridging the gap between pilot success and routine clinical use will require a coordinated effort across these stakeholder groups.

## Clinical Relevance Statement

This paper illustrates how patient-centeredness must be accounted for in the full sociotechnical landscape of digital technologies that support patients in making decisions about their health care. Drawing on lessons learned from implementing and evaluating multiple pilots, we highlight how patient-centered priorities informed key design choices and shaped broader implementation strategies. The paper identifies considerations for clinicians, health system leaders, and policymakers to enable sustained adoption, including designing for patient engagement, enhancing interoperability, establishing new monitoring workflows for patient-contributed data, and demonstrating financial sustainability.

## Multiple-Choice Questions

1. Why was early and continuous patient codesign important for the adoption of patient engagement technologies?
  - It ensured compliance with institutional review requirements.
  - It reduced the need for clinician training during implementation.
  - It aligned technology design with patients' routines and preferences.
  - It eliminated the need for postimplementation usability testing.
  **Correct Answer** : The correct answer is option c. The article highlights that early and ongoing patient codesign aligns technology design with patients' lived experiences, including daily routines, communication preferences, and technology comfort levels. Codesign activities such as interviews, think-aloud sessions, and surveys led to meaningful design changes—such as message tone and timing. Patient codesign did not eliminate the need for training or postimplementation evaluation, nor was it driven by regulatory requirements; instead, it ensured that technologies fit into patients' lifeflows.
2. What most limited the scalable integration of patient contributed data into electronic health records across the evaluated pilots?
  - Limited availability of patient generated data sources.
  - Uneven adoption of syntactic and semantic standards.
  - Insufficient clinician interest in patient reported outcomes.
  - Lack of national policies supporting interoperability.
  **Correct Answer** : The correct answer is option b. Although syntactic and semantic standards exist, the article shows that uneven adoption and implementation of these standards across EHR vendors and health systems was a persistent barrier to scalable data integration. Many pilots encountered gaps in support for patient-reported data types, such as symptoms, requiring site-specific workarounds, or custom builds that undermined interoperability.
3. How does the expanded sociotechnical model proposed in this paper differ from traditional applications of the model?
  - It prioritizes organizational efficiency over patient experience
  - It incorporates patients' daily contexts and technology use outside clinical settings
  - It replaces clinician workflows with automated decision support
  - It focuses primarily on national policy and regulatory drivers
  **Correct Answer** : The correct answer is option b. Traditional applications of the Sociotechnical Model of Health Information Technology focus largely on technology use within health care delivery organizations, emphasizing interactions among clinicians, IT systems, workflows, and policies. In contrast, this paper expands the sociotechnical lens to explicitly include how patients engage with technologies in their everyday lives outside clinical settings. The expanded model recognizes that adoption and sustainability depend not only on organizational readiness but also on how well technologies align with patients' daily contexts.
